# Intelligent Gesture Recognition Based on Screen Reflectance Multi-Band Spectral Features

**DOI:** 10.3390/s24175519

**Published:** 2024-08-26

**Authors:** Peiying Lin, Chenrui Li, Sijie Chen, Jiangtao Huangfu, Wei Yuan

**Affiliations:** 1 School of Electrical and Information Engineering, Jiangsu University of Science and Technology, Zhangjiagang 215600, China; yuanwei@just.edu.cn; 2 Laboratory of Applied Research on Electromagnetics, Zhejiang University, Hangzhou 310027, China; 3210103050@zju.edu.cn (C.L.); 22331025@zju.edu.cn (S.C.); huangfujt@zju.edu.cn (J.H.)

**Keywords:** multi-band spectra, human–computer interaction, gesture recognition

## Abstract

Human–computer interaction (HCI) with screens through gestures is a pivotal method amidst the digitalization trend. In this work, a gesture recognition method is proposed that combines multi-band spectral features with spatial characteristics of screen-reflected light. Based on the method, a red-green-blue (RGB) three-channel spectral gesture recognition system has been developed, composed of a display screen integrated with narrowband spectral receivers as the hardware setup. During system operation, emitted light from the screen is reflected by gestures and received by the narrowband spectral receivers. These receivers at various locations are tasked with capturing multiple narrowband spectra and converting them into light-intensity series. The availability of multi-narrowband spectral data integrates multidimensional features from frequency and spatial domains, enhancing classification capabilities. Based on the RGB three-channel spectral features, this work formulates an RGB multi-channel convolutional neural network long short-term memory (CNN-LSTM) gesture recognition model. It achieves accuracies of 99.93% in darkness and 99.89% in illuminated conditions. This indicates the system’s capability for stable operation across different lighting conditions and accurate interaction. The intelligent gesture recognition method can be widely applied for interactive purposes on various screens such as computers and mobile phones, facilitating more convenient and precise HCI.

## 1. Introduction

In contemporary society, digitization has emerged as a crucial trend, fundamentally transforming social dynamics. The widespread development of digital technologies and the growing presence of smart devices are seamlessly integrating into our daily lives [1]. This transformation is facilitated by HCI, which is a discipline focused on designing, evaluating, and implementing interactive computing systems for human use as well as studying the fundamental phenomena [2]. Due to its close collaboration and interaction with users, HCI has become a core area for enhancing the usability of digital devices [3]. Central to visualizing information in this interaction is the display device, which plays a crucial role in data communication and allows for intuitive user interactions [4].

Traditional methods of interaction with display devices rely on desktop setups equipped with keyboards and mice [5]. With technological advancements, more flexible and convenient methods have been adopted. Touch interaction is widely embraced due to its direct method of intuitive control and data transmission [6], enabling the transfer of complex information such as multi-touch [7,8] or multi-user collaboration [9]. Compared with tactile modes, voice interaction eliminates the need for direct physical contact. For instance, in driving scenarios, non-contact voice interaction proves more user-friendly [10], thereby enhancing user satisfaction [11]. However, voice input prolongs interaction response time [12] and is constrained in environments with background noise [13]. Computer vision enables diverse interactions involving facial [14] and bodily gestures [15], but its accuracy depends on the resolution and frame rate of the camera [16]. According to the World Health Organization (2024), over 466 million people worldwide suffer from severe hearing loss. Gesture-based interaction offers a promising solution for enhancing communication for these individuals [17]. Technologies such as computer vision [18,19,20], audio [21], and radar detection [22,23] enable gesture-based screen interactions. Feature extraction combined with detection [18,19,20,21,22,23,24,25,26,27,28,29,30,31,32,33,34] is commonly used in gesture recognition. These features include frequency [21], motion [23], skin color [26], skeletal structure [27], and shape [28], as well as spatio-temporal features [29,30,31] derived from deep networks. Additionally, depth information [32,33] and optical flow [34] are frequently utilized to supplement image data, although this demands more advanced equipment. By combining various features and employing multi-stream techniques, it is possible to achieve more effective feature fusion [35].

The method of light-signal-based interaction offers an alternative non-contact solution. For instance, in medical applications [36], touch-based interaction screens increase the risk of surgical infections. Furthermore, visual and voice interactions require the collection of biological information, which compromises privacy and security. Therefore, infrared laser positioning can be employed as an alternative. Infrared spectra can also be specifically applied in human signal measurement [37]. In addition to infrared technology, industry and the research community have developed numerous visible-light positioning (VLP) systems [38] and visible-light sensing (VLS) systems [39], which require commonly used light emitting diodes (LED) as lighting sources and light sensors to form the systems [40]. Similarly, utilizing visible light for screen sensing involves using ambient light sensors to capture light intensity information from external light sources at various angles relative to the screen [41]. Combining light sensing with gesture interaction provides a convenient and secure method for non-contact interaction [42,43].

This study introduces a gesture recognition approach that combines multi-band spectral features with the spatial characteristics of screen-reflected light. In this approach, display screens are used as light sources for illumination, and various gestures produce unique patterns of reflected spectra in front of the screen. Concurrently, multiple narrowband spectral receivers capture data across multi-band spectra. This combination of spectral data is fused with spatial information, enabling the formation of comprehensive multidimensional features essential for accurate gesture recognition. One of the key advantages of this screen interactive system is its independence from the additional light sources, radar systems, or camera devices commonly used for similar purposes. Moreover, the implementation of cost-effective narrowband spectral receivers enhances affordability without compromising performance. Additionally, this approach addresses privacy concerns by minimizing the collection of biometric information, ensuring a secure and user-friendly interaction environment.

The remainder of the paper is structured as follows: Section 2 introduces a gesture recognition method based on multi-band spectral features, implementing an RGB three-channel narrowband spectral gesture recognition system. Section 3 outlines the data collection process. Section 4 details the RGB multi-channel CNN-LSTM gesture recognition model. Then, the experimental results are presented and discussed in Section 5. Finally, Section 6 serves as the conclusion of this paper.

## 2. Principles and System

### 2.1. Principles

**Figure 1:**
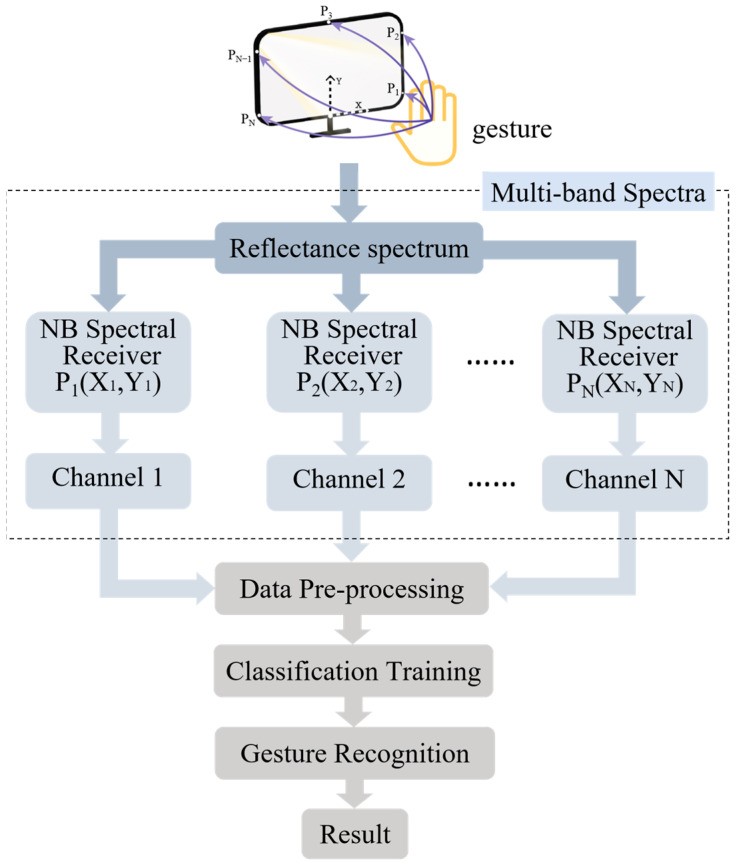
Flowchart of the gesture recognition method based on multi-band spectral features.

A gesture recognition method based on multi-band spectral features is proposed in this work, which combines the spectral and spatial characteristics of screen light reflected from gestures. The specific process is illustrated in Figure 1. The intelligent gesture recognition system according to this method mainly consists of a light-emitting display screen and a plurality of narrowband spectral receivers. The system works by orienting the target gesture toward the screen, in which the screen serves the purpose of providing illumination on the gesture while displaying normally. The light information reflected by the gesture is captured by multiple narrowband spectral receivers mounted on the screen. These receivers are installed at different positions on the plane where the screen is located, and the reflected light from the gestures generates different spectral distributions in various spatial locations. Furthermore, these receivers capture narrowband spectra from different bands and convert them into photonic signals to obtain light-intensity measurements. As a result, spectral data containing various bands from different coordinates can be received, which provides the possibility to train different characteristics in frequency and spatial domains. Based on the measurements from multiple receivers and combined with classification algorithms, different gestures can be effectively classified, significantly improving classification efficiency and recognition accuracy.

### 2.2. System

**Figure 2:**
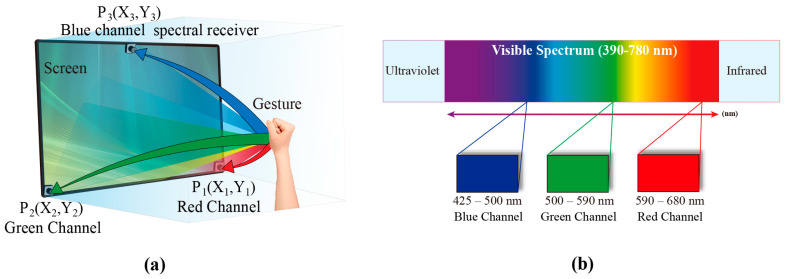
(**a**) RGB three-channel narrowband spectral gesture recognition system; (**b**) filtered RGB three-channel narrowband spectra from the system.

According to the method, an RGB three-channel narrowband spectral gesture recognition system is realized as shown in Figure 2a, with three narrowband spectral receivers installed at different coordinates of the screen plane. The light emitted from the screen is reflected by the gestures and then captured by receivers positioned at three coordinates on the screen: bottom-right, bottom-left, and top-center. These receivers record narrowband spectral data corresponding to the red, green, and blue channels, as in Figure 2b. Consequently, the three-channel data incorporate both spectral and spatial information features.

**Figure 3:**
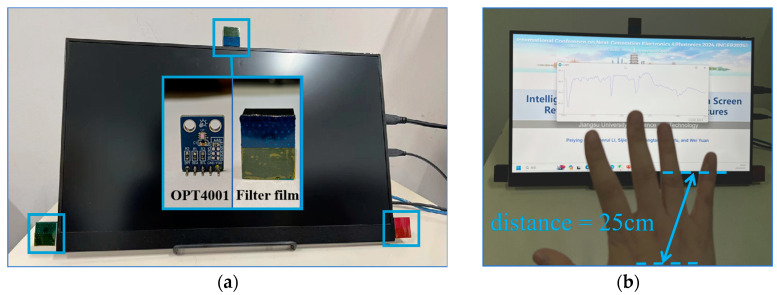
(**a**) The photograph of the system configuration; (**b**) the data fluctuations of the light intensity reflecting hand gesture variation.

**Figure 4:**
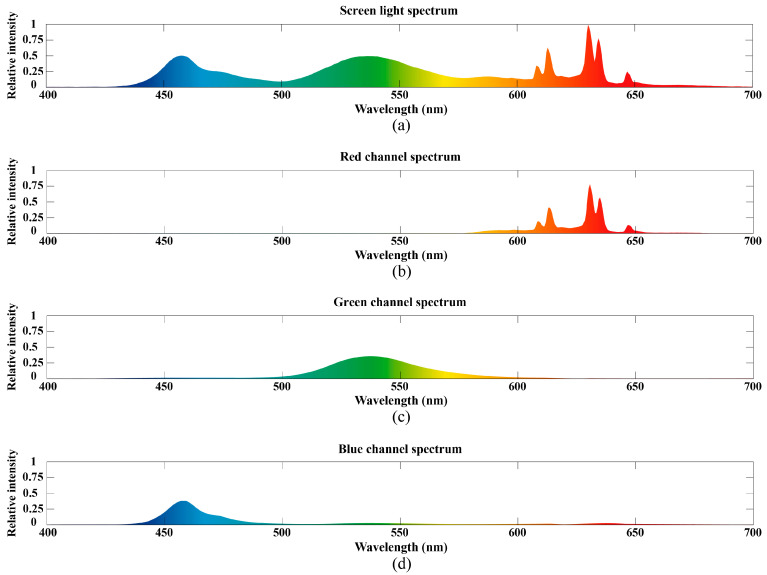
Spectral filtering effects of the filter films on display light measured by the spectrometer: (**a**) The spectrum measured by the spectrometer when the screen emits white light; (**b**) the spectrum through the red channel narrowband filter; (**c**) the spectrum through the green channel narrowband filter; (**d**) the spectrum through the blue channel narrowband filter.

The system configuration is set up as in Figure 3a, with a screen size of 17.2 inches. The narrowband spectral receiver consists of a light sensor and a filter film. The light sensor chip is OPT4001, with a measurement range of 1–918 lux and an accuracy of up to 112 millilux. The data sampling rate is set at 100 Hz in the experiments. The filter film is placed in front of the light sensor to selectively receive specific wavelength light. Figure 4 illustrates the spectral filtering effect of the filter films measured by the spectrometer on the screen light, with Figure 4a depicting the measured spectrum when the screen emits white light. Figure 4b–d depict the corresponding RGB narrowband spectra after passing through the filter films. The RGB spectral wavelengths received through the filter films are 590–680 nm, 500–590 nm, and 425–500 nm, respectively. The filter effectively filters out spectra outside the narrow band without affecting the shape and characteristics of the target narrowband spectra, while also reducing the intensity of the entire spectrum. The use of light-intensity sensors as spectral receivers not only enhances the sensitivity of light detection but also provides convenience and cost reduction compared with spectrometers. The spectral receiver outputs a time series of integrated light intensity corresponding to each narrowband spectrum.

During system operation, the display screen emits light normally. When a person’s hand is placed within a distance range of 10–70 cm directly in front of the screen, the screen light reflected by gestures reaches the narrowband spectral receivers positioned at different spatial locations. After passing through the filter film, only light of specific wavelengths is allowed to be received by the light-intensity sensor. The receiver converts narrowband spectral information into light-intensity time series, which are transmitted to the screen control terminal. The intensity information is visualized in real time on the screen. Variations in gestures cause changes in the reflected light intensity, which can be observed as corresponding data fluctuations on the screen in Figure 3b, reflecting changes in hand movements.

## 3. Data Collection

**Figure 5:**
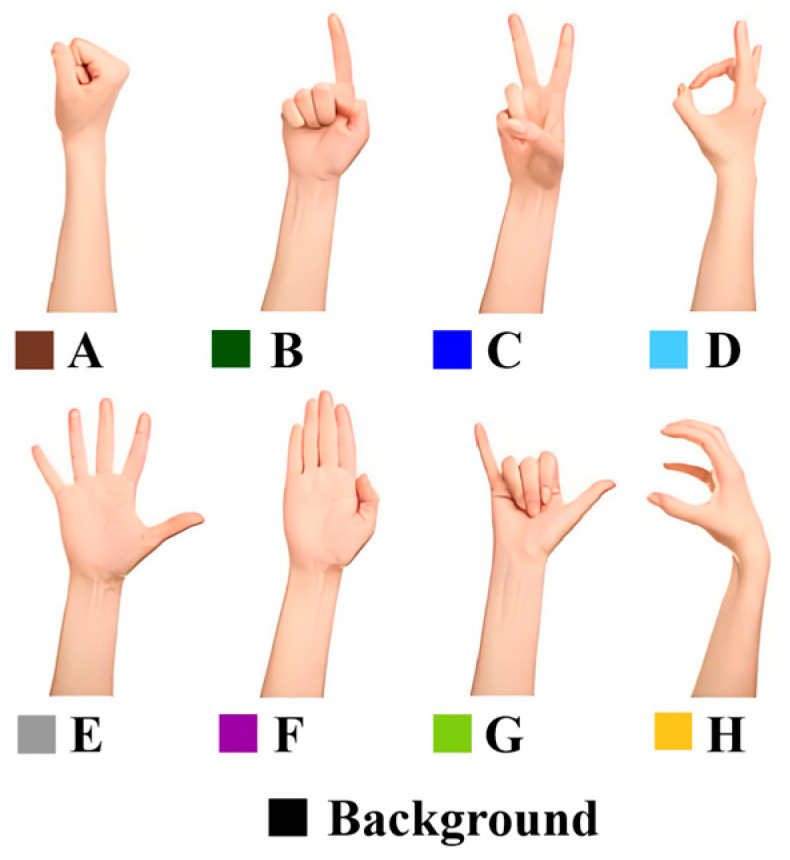
Eight gestures in the gesture recognition.

Section 2 of the system is designed for implementing gesture-based HCI, applied in eight gestures as depicted in Figure 5, each annotated with a distinct color. The term “Background” refers to the scenario where no gesture is present in front of the screen, serving as a baseline control. The process of data collection is conducted through the RGB three-channel spectral receivers of the system setup, with the datasets structured into two main groups, labeled Dataset 1 and Dataset 2.

**Figure 6:**
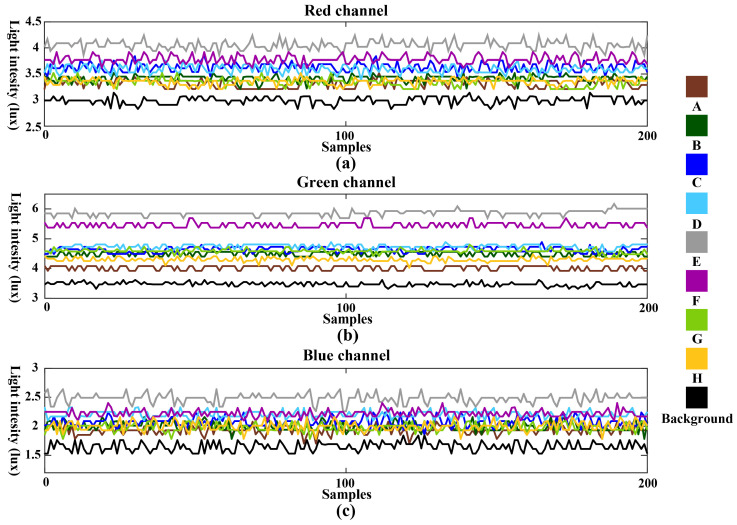
The light intensity data from RGB three-channel spectral receivers under dark conditions for the eight gestures and control group: (**a**) data from the red channel; (**b**) data from the green channel; (**c**) data from the blue channel.

In Dataset 1, data collection involved performing eight gestures directly facing the screen in darkness, with the screen display being the only light source. Under identical display conditions, Figure 6 illustrates the light intensity data from RGB three-channel spectral receivers for the eight gestures and control group. In this dataset, variations in light intensity stem from changes in the distribution of screen-reflected light caused by the gestures. The lowest light intensity occurs when no gesture is present, which aligns with the operational principle of the system. Moreover, Figure 6 presents notable differences in data distribution across different channels receiving the same display content, indicating varying impacts of gestures on the light-intensity information received by each channel. These differences arise from spatial information and spectral wavelength variations across the channels. The multi-channel data constitute multidimensional time-series features, essential for accurate gesture recognition.

**Figure 7:**
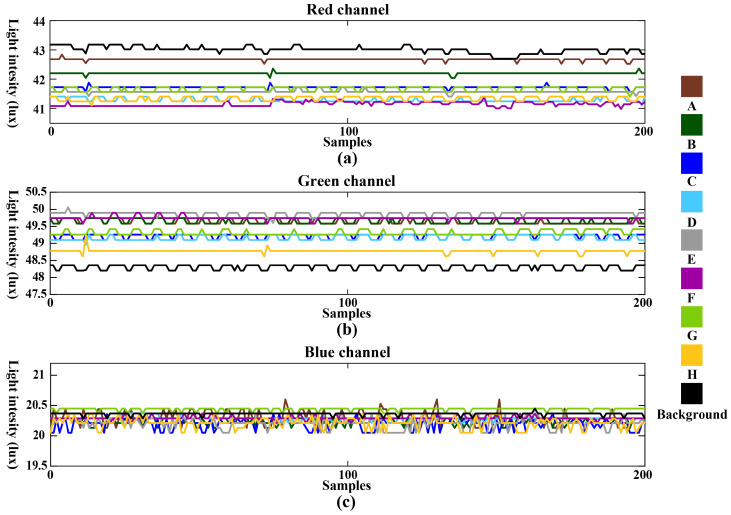
The light intensity data from RGB three-channel spectral receivers under ambient light conditions for the eight gestures and control group: (**a**) data from the red channel; (**b**) data from the green channel; (**c**) data from the blue channel.

In Dataset 2, data corresponding to eight gestures were collected under ambient light conditions. The purpose of this group of data collection is to test the influence of ambient light on gesture recognition accuracy. The ambient light source is a commonly used PWM modulated ceiling lamp, with an average light intensity of 65 lux measured by narrowband spectral receivers. The light-intensity data for the eight gestures collected from the RGB three channels, as well as the control group, are shown in Figure 7. Variations in different gestures not only affect changes in screen-reflected light but also influence the reception of ambient light by the narrowband spectral receivers. Consequently, the changes in light intensity captured by the narrowband spectral receivers integrate the effects of both factors. The impact of gestures on ambient light comprises reflections and obstruction of light caused by the gestures, with the proportion depending on spatial positioning and spectral wavelength. For instance, in the green channel, the light intensity data for the control group without gestures are lowest, indicating a significant effect of gestures on the reflection of green light. Conversely, in the red channel, the data show the opposite trend, with the light intensity for the control group without gestures being highest, indicating a greater impact of gestures on obstructing red light. The blue channel data exhibit a more balanced effect from both factors, resulting in less discernible features visually. Therefore, in Dataset 2 as shown in Figure 7, the complex lighting conditions lead to more pronounced differences in the distribution of data across different channels. Such complex illumination environments necessitate the integration of spatial information and multi-band narrowband spectra to capture multidimensional features effectively, thereby enhancing gesture recognition accuracy.

During the data collection process for both datasets, each gesture sample was captured for 1 s, with each sample containing 100 samplings. The gesture data were sourced from 10 volunteers, comprising 5 men and 5 women. Variations in their hand sizes and skin tones resulted in different effects on the reflected spectrum. During collection, each volunteer’s hand was positioned 30 cm from the screen, with each gesture from each individual being sampled 92 times, corresponding to 92 different images with various color tones displayed on the screen. Each dataset consisted of 920 samples per gesture, totaling 920 × 8 data points, as shown in Table 1. A randomly selected quarter of the dataset was designated as the test set.

Normalization was applied to the collected data before analysis. This process transformed the time series of each sample into a one-dimensional matrix, ensuring values ranged between −1 and 1. The normalization formula is as follows:

(1) $$x=-1+\frac{x-x_{min}}{x_{max}-x_{min}}\times 2$$

## 4. RGB Multi-Channel CNN-LSTM Gesture Recognition Model

**Figure 8:**
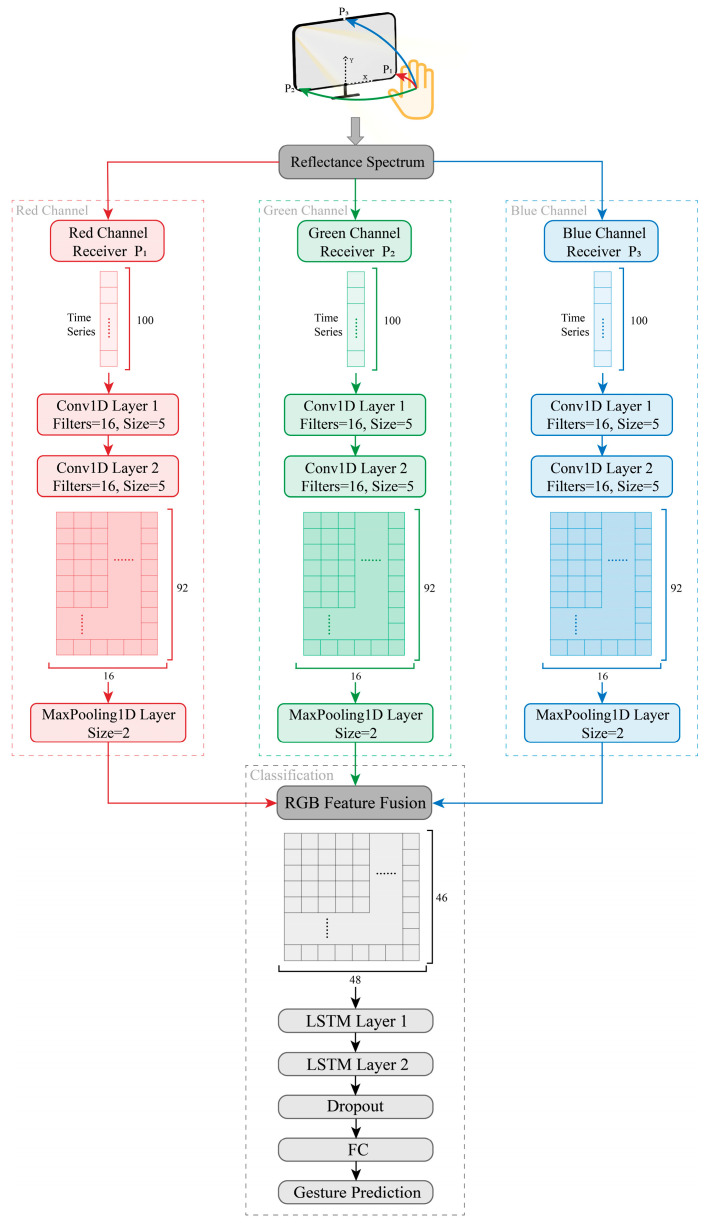
The RGB multi-channel CNN-LSTM gesture recognition model.

This section presents a gesture recognition model that integrates RGB multi-channel 1-dimensional convolutional neural network (1D-CNN) and LSTM architectures. The model processes RGB three-channel light intensity time series as the input and generates gesture classification predictions as the output, as depicted in Figure 8. Initially, RGB multi-channel 1D-CNN is employed to extract multidimensional features from the input time-series data. Subsequently, these feature sequences are fed into LSTM for gesture classification. This hybrid approach effectively harnesses the feature extraction capabilities of 1D-CNN and the sequence modeling capabilities of LSTM. It synergizes with the multi-channel spectral information acquisition capability of the system hardware in this work, enabling accurate gesture-based HCI.

### 4.1. RGB Three-Channel 1D-CNN Feature Extractor

**Figure 9:**
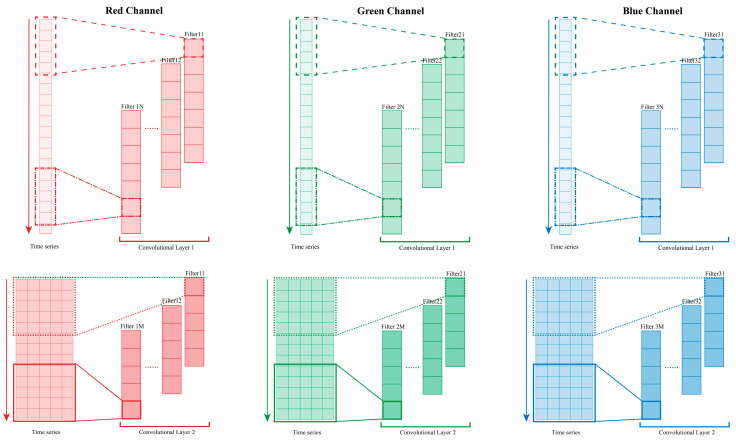
The feature extraction process of RGB multi-channel 1D-CNN.

CNN can serve as a feature extractor [44], specifically, employing 1D-CNN for the analysis of time-series data from sensors. Accordingly, two layers of 1-dimensional convolution (Conv1D) are employed to extract multidimensional features from RGB three-channel data. Figure 9 illustrates the process of extracting time-series features. Each sample input to the gesture recognition model consists of 100 data points sampled over 1 s. Filters convolve with the time series to extract features. Each Conv1D layer incorporates 16 filters with a window size of 5, sliding down the data with a default stride of 1. The input data comprise RGB three channels, forming a 100 × 3 matrix that corresponds to the three channels in the Conv1D layers. Each channel shares the same structure but employs different filter combinations based on the data characteristics, thus enhancing the representation of the input time-series features. Finally, following a 1-dimensional max pooling (MaxPooling1D) layer with a size of 2, the features are merged across multiple channels as shown in Figure 8, resulting in each sample being represented as a multidimensional feature sequence of size 46 × 48 matrix.

### 4.2. LSTM Network

**Figure 10:**
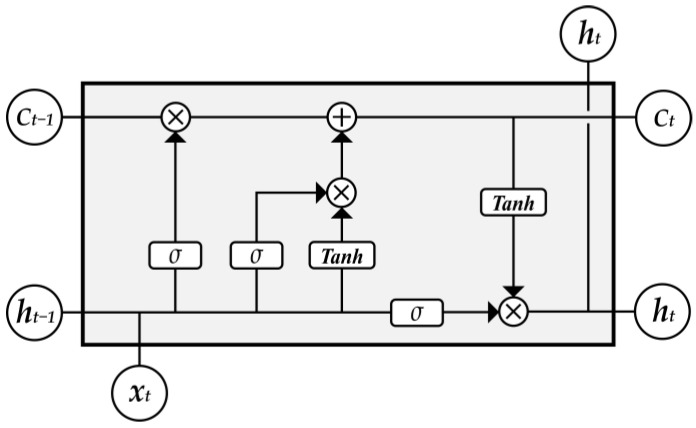
Network structure of LSTM.

LSTM networks [45], a specialized category of recurrent neural networks (RNNs), are proficient in identifying and forecasting both short-term and long-term dependencies within time-series data [46]. Information is transmitted among different cells of the hidden layer through several controllable gates [47], as depicted in Figure 10. The symbol c represents the memory cell state. The network contains input gate $i_{t}$, output gate $o_{t}$, and forget gate $f_{t}$. The input gate $i_{t}$ determines the contributions of the input data at time step t for updating the memory cell, while the forget gate $f_{t}$ determines how much of the last moment’s cell $c_{t-1}$ is retained for the current state $c_{t}$. The output gate $o_{t}$ controls how much information is output for cell status. Finally, ${\tilde{c}}_{t}$ represents the next state. The LSTM network updates its information through the following Equations (2)–(9):

(2) $$i_{t}=\sigma_{i}(W_{i}\cdot {[h_{t-1},\;x}_{t}]+b_{i})$$

(3) $$o_{t}=\sigma_{o}(W_{o}\cdot {[h_{t-1},\;x}_{t}]+b_{o})$$

(4) $$f_{t}=\sigma_{f}(W_{f}\cdot {[h_{t-1},\;x}_{t}]+b_{f})$$

(5) $${\tilde{c}}_{t}=tanh(W_{c}\cdot {[h_{t-1},\;x}_{t}]+b_{c})$$

(6) $${\;c}_{t}={f_{t}⊙c}_{t-1}+i_{t}⊙{\tilde{c}}_{t}$$

(7) $$h_{t}=o_{t}⊙tanh(c_{t})$$

(8) $$sigmoid\left(x\right)=\frac{1}{1+e^{-x}}$$

(9) $$\tanh\left(x\right)=\frac{e^{x}-e^{-x}}{e^{x}+e^{-x}}$$

where $W_{i}$, $W_{o}$, $W_{f}$, and $W_{c}$ represent the input weights; $b_{i}$, $b_{o}$, $b_{f}$, and $b_{c}$ represent the bias weights; ⊙ denotes element-wise product; σ represents the sigmoid function as Equation (8), and the hyperbolic tangent function is illustrated in Equation (9); and $h_{t}$ represents the output. The classifier consists of two layers of LSTM, one dropout layer and one fully connected (FC) layer, and finally, uses softmax activation to output gesture labels. Training is conducted using the Adam optimizer with a learning rate of 0.001, a batch size of 27, and 128 nodes in the hidden layers. The model was developed and trained on the Anaconda3 platform, utilizing an NVIDIA GeForce RTX 3070 GPU.

### 4.3. Evaluation

In this work, macro averaging [48] is employed to evaluate the performance metrics of the multi-class classification model, including accuracy, precision, recall, and F-score [49]. These metrics are expressed by the following Formulas (10)–(13), where TP = true positives, FP = false positives, FN = false negatives, and TN = true negatives. Accuracy is the most used empirical measure, which is the ratio of the number of correct predictions to the total number of predictions.

(10) $$Accuracy=\frac{TP+TN}{TP+FP+FN+TN}$$

Precision is the ratio of the correct positive predictions to the total number of predictions as positives.

(11) $$Precision=\frac{TP}{TP+FP}$$

Recall is the ratio of the correct positive predictions to the total number of positive instances, also known as sensitivity.

(12) $$Recall=\frac{TP}{TP+FN}=Sensitivity$$

F-score is the harmonic mean of the precision and recall, evenly balanced when β=1. Higher values of the F-score indicate a better balance between precision and recall.

(13) $$F\text{-}score=\frac{(\beta^{2}+1)*Precision\times Recall}{\beta^{2}*Precision+Recall}$$

## 5. Experimental Results and Discussion

The experiments on gesture recognition are divided into two steps, labeled as Experiment I and Experiment II, applied separately to Dataset 1 and Dataset 2. Each dataset comprises 5520 samples for training and 1840 samples for testing. Experiment I evaluates the performance of the gesture recognition system under dark conditions using only screen-reflected spectra. Experiment II assesses the performance in the presence of ambient light sources, considering the combined effects of screen-reflected light and external illumination.

### 5.1. Experimental I Results

**Figure 11:**
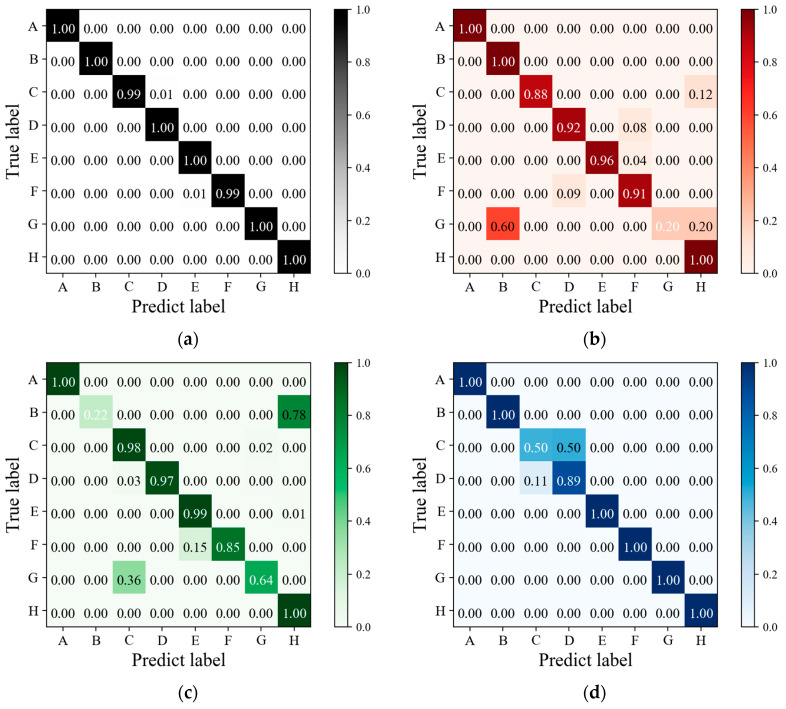
The confusion matrix results of Experiment I: (**a**) The confusion matrix result of RGB three-channel gesture classification; (**b**) the confusion matrix result of red-channel gesture classification; (**c**) the confusion matrix result of green-channel gesture classification; (**d**) the confusion matrix result of blue-channel gesture classification.

Figure 11 presents the confusion matrix results for Experiment I evaluated on Dataset 1. Figure 11a depicts the confusion matrix for RGB three-channel gesture classification, indicating an accuracy of 99.93%, with accuracies exceeding 99% for all eight gestures. Detailed performance metrics are listed in Table 2, where the precision, recall, and F1-score of this classification model all achieve 99.73%. To demonstrate the efficacy of multi-band spectral features in enhancing gesture recognition, the classification results of Dataset 1 are compared between the RGB three-channel and single-channel. The single-channel classification employs data from either the red, green, or blue channel, based on the single-channel CNN-LSTM gesture recognition model. Figure 11b–d show the confusion matrices for the red, green, and blue channels, respectively, with accuracies of 96.45%, 95.82%, and 98.07%. Results from Table 2 indicate inferior metrics for precision, recall, and F1-score in the single-channel classification, highlighting the superior performance of the multi-channel classification model across all metrics compared with the single-channel classification models.

**Figure 12:**
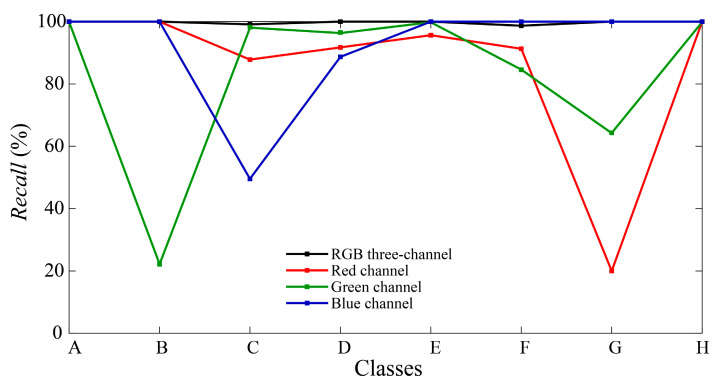
The recall results for each class across different classification models in Experiment I.

For a clearer comparison, Figure 12 displays the recall results for each class across the different classification models. Recall assesses the classifier’s ability to correctly identify all positive instances [49]. Analysis of the curves in Figure 12 reveals varying effectiveness of different channels in recognizing each gesture. For example, the classification model trained on the red channel performs poorly for gesture G due to similarities in light intensity with gesture B, as observed in the sampling data of Figure 6, resulting in misclassification of G. Similarly, the green channel shows inadequate recognition of gesture B. In the blue channel, gestures C and D exhibit frequent confusion while demonstrating robust performance for other gestures. The disparate recognition performances across single channels highlight distinct spectral characteristics. Screen-reflected light for the same gesture exhibits spectral variation across different coordinates and is captured by diverse narrowband receivers, further differentiating the data from each channel. Additionally, features of the single channels are limited, leading to notably poorer recognition of specific gestures. In contrast, the multi-channel classification model mitigates these challenges by combining RGB three-channel spectral data from different spatial coordinates, thereby improving the accuracy of gesture recognition. In conclusion, the integration of multi-band spectral features with spatial information markedly enhances the accuracy of gesture recognition.

### 5.2. Experimental II Results

**Figure 13:**
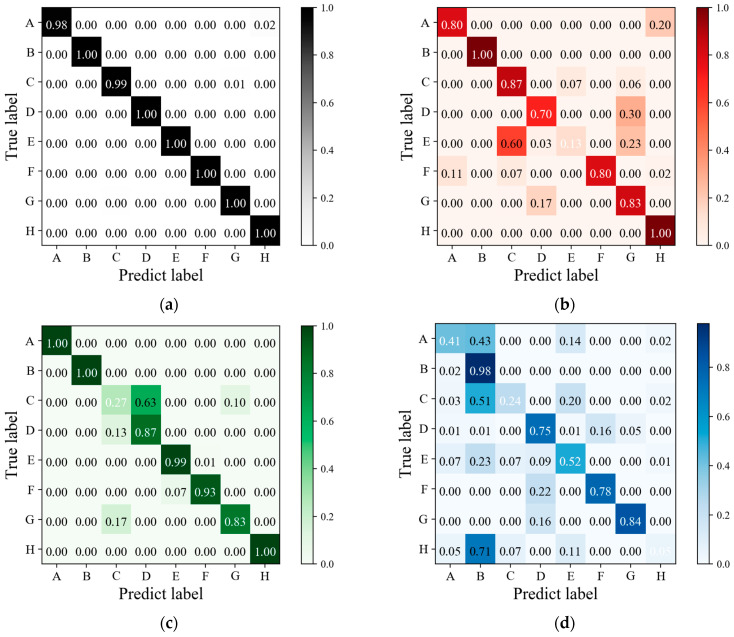
The confusion matrix results of experiment II: (**a**) The confusion matrix result of RGB three-channel gesture classification; (**b**) the confusion matrix result of red-channel gesture classification; (**c**) the confusion matrix result of green-channel gesture classification; (**d**) the confusion matrix result of blue-channel gesture classification.

Figure 13 illustrates the confusion matrix results of Experiment II evaluated on Dataset 2. Metrics for all classification models are listed in Table 2. The confusion matrix for the RGB three-channel classification model is shown in Figure 13a. Despite the more complex composition of light sources in Experiment II, the classification results remain highly accurate, with an accuracy of 99.89%. The model achieves precision, recall, and F1-score metrics of 99.57%, indicating that the proposed gesture recognition method and system can operate effectively even in the presence of external light sources.

**Figure 14:**
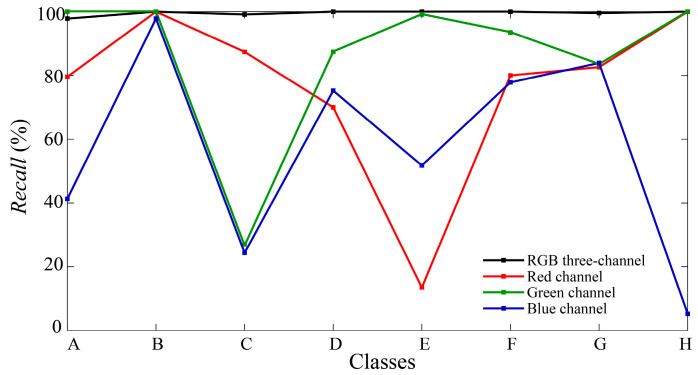
The recall results for each class across different classification models in Experiment II.

Additionally, this step of the experiment also evaluates the classification of single-channel data. Figure 13b–d depict the confusion matrices for the red, green, and blue channels, respectively, with accuracies of 94.16%, 96.56%, and 89.29%. Results from Table 2 demonstrate lower performance metrics for the single-channel classification, underscoring a substantial disparity when compared with the multi-channel classification model. Recall results for each class are compared in Figure 14, revealing that the red channel model performs poorly in recognizing gesture E, the green channel struggles with gesture C, and the overall classification performance in the blue channel is inadequate. Based on the analysis of light intensity data in Figure 7, in the presence of ambient light, narrowband spectral receivers integrate light information affected by gesture interaction with both screen light and ambient light. Different spatial positions and spectral wavelengths influence single-channel performance differently: the green channel is mainly influenced by reflected light, the red channel by shadows of ambient light caused by gestures, and the blue channel by varying light intensity due to both reflected light and shadows from gestures. Consequently, the characteristics captured by the blue channel are not sufficiently distinct, resulting in poor classification performance. In such complex lighting environments, the integration of multi-band spectral data from multiple spatial positions becomes crucial. Even in cases where individual single-channel classifications perform poorly, such as gesture C, with recall values of 87.39%, 26.52%, and 24.35% in the red, green, and blue channels, respectively, the combination of spatial and spectral features with a multi-channel gesture recognition model effectively raises the recall of gesture C to 99.13%. This synergistic effect demonstrates how the combined utilization of multiple channels yields superior performance compared with each channel individually.

### 5.3. Discussion

Based on the results above, we summarize the experimental results and discuss future directions for improvements.

The experiments validated the proposed gesture recognition method that integrates multi-band spectral data with spatial information. Experiment I, conducted in darkness, demonstrated high accuracy of 99.93% using only screen-reflected spectra for gesture recognition. In Experiment II, which introduced ambient light sources in complex lighting environments, single-channel recognition performed poorly. In contrast, the proposed multi-band spectral gesture recognition model maintained effective performance, significantly enhancing recognition accuracy to 99.89% compared with the single-channel models. The system is well-suited for indoor applications.

We compared the experimental results with other recent non-contact screen interaction systems that employ gesture recognition, as listed in Table 3. In similar research, computer vision [19,20] is commonly used, with performance dependent on image quality and camera specifications. Passive sound sensing [21] is another convenient gesture interaction method but, like computer vision, it faces privacy and security concerns. Cheng et al. [23] developed a radar-based system with high recognition accuracy, though it incurs significant equipment costs. In the design by researchers Liao et al. [42], a single light sensor was installed on the screen, necessitating coordination with the display content and leaving room for further system improvements. Our proposed system offers advantages, including lower cost and easier portability of the narrowband spectral receivers. It addresses privacy and security concerns while achieving a relatively high level of recognition accuracy. However, the limitation lies in the restricted range of recognizable gestures and scenarios. We propose the following directions for improvements.

(1) The gesture categories and spectral ranges in this work are limited. In future research, expanding the range of gesture classifications could enable more complex human–machine interactions, potentially incorporating dynamic movements. For example, integrating with a sign language database would greatly enhance the system’s practicality for individuals with hearing and speech impairments. To achieve this, detailed plans for data collection and window segmentation will be essential. Additionally, this work focused solely on collecting spectral data within the visible light range. Future extensions could involve expanding to wider spectral ranges to fully leverage the data characteristics across different spectra.
(2) This work established an RGB three-channel narrowband spectral gesture recognition system. Future efforts will focus on optimizing the reception system to advance the accuracy and applicability of the proposed method in diverse real-world scenarios. To enhance accuracy in complex interactions, deploying more narrowband receivers at multiple locations to establish a reception matrix would prove beneficial.

## 6. Conclusions

The intelligent gesture recognition method proposed in this paper leverages multi-band spectral features that integrate frequency domain and spatial domain information to enhance accuracy. Based on this method, an RGB three-channel narrowband spectral gesture recognition system is developed, incorporating a screen and multiple narrowband spectral receivers as essential hardware components. Integrated with the RGB multi-channel CNN-LSTM classification model, the system accurately recognizes eight types of gestures and enables interaction with display screens. It processes multi-channel time series data from narrowband spectral receivers, achieving accuracies of 99.93% in dark conditions and 99.89% in illuminated conditions. The collaborative effect of the multi-channel features enhances performance, significantly improving recognition accuracy compared with single-channel models. This gesture recognition method offers straightforward implementation, ensuring privacy and security and facilitating its widespread application in various screen-based human–machine interactions.

## Figures and Tables

**Table 1:** Description of the datasets.

Dataset	Light Source	Volunteers	Number of Gestures	Samples
1	Screen	10	8	920 × 8
2	Screen + ambient light	10	8	920 × 8

**Table 2:** Evaluation metrics of the classification models in experiments.

Experiment	Channel	Accuracy	Precision	Recall	F1-Score
I	RGB three-channel	99.93%	99.73%	99.73%	99.73%
	Red channel	96.45%	89.66%	85.82%	83.59%
	Green channel	95.82%	88.94%	83.26%	81.43%
	Blue channel	98.07%	93.15%	92.28%	91.98%
II	RGB three-channel	99.89%	99.57%	99.57%	99.57%
	Red channel	94.16%	78.53%	76.63%	74.42%
	Green channel	96.56%	85.94%	86.25%	85.32%
	Blue channel	89.29%	63.62%	57.17%	54.45%

**Table 3:** Comparison with other recent non-contact screen interaction systems based on gesture recognition.

System	Equipment	Accuracy	Number of Gestures	Algorithm
Zahra et al. [19]	Camera	93.35%	6	Skin detection and genetic algorithm
Benitez-Garcia et al. [20]	Camera	85.10%	13	Temporal segment networks (TSN), temporal shift modules (TSM)
Luo et al. [21]	Microphone	93.20%	7	Feature extraction and support vector machine (SVM)
Cheng et al. [23]	Millimeter wave radar and a thermal imager	100.00%	5	Feature extraction and gated recurrent unit (GRU)
Liao et al. [42]	Ambient light sensor	96.10%	9	Feature extraction and k-nearest neighbors (KNN)
This work	Narrowband spectral receivers	99.93%	8	RGB multi-channel CNN-LSTM

## Data Availability

Data are contained within the article.
